# Ruhigstellungstechniken der oberen Extremität bei Kindern

**DOI:** 10.1007/s00064-025-00896-8

**Published:** 2025-04-14

**Authors:** Nadine Kaiser, Teddy Slongo

**Affiliations:** Abteilung für Kinderorthopädie/Kindertraumatologie, Kinderchirurgische Universitätsklinik, Inselspital, Freiburgstr., 3010 Bern, Schweiz

**Keywords:** Unterarmfraktur, Ellbogenfraktur, Kindesalter, Konservative Behandlung, Gips, Forearm fracture, Elbow fracture, Child, Conservative treatment, Plaster cast

## Abstract

**Behandlungsziel:**

Konservative Behandlung von stabilen Frakturen der oberen Extremität bei Kindern.

**Indikationen:**

Undislozierte und altersentsprechend tolerabel dislozierte/angulierte Frakturen der Hand, des Vorderarmes und des Ellbogens, bei denen nach Reposition nicht die Gefahr einer sekundären Instabilität besteht.

**Kontraindikationen:**

Offene Frakturen.

**Behandlungsmöglichkeiten:**

***Unterarmschiene/Unterarmgips*** bei stabilen Verletzungen des distalen Radius oder der distalen Ulna.

***Oberarmschiene/Oberarmgips*** bei Verletzungen von Radius und Ulnaschaft und nach reponierten Unterarmschaftfrakturen sowie bei stabilen, undislozierten Verletzungen des Ellbogens.

***Intrinsic*** ***+*** *Schiene* bei Verletzungen der Langfinger und Mittelhand.

**Weiterbehandlung:**

Bei stabilen Verletzungen Ruhigstellung zur Analgesie für 3 bis 4 Wochen. Klinische Kontrolle mit Freigabe der Beweglichkeit.

Bei reponierten Frakturen oder im Rahmen der Spontankorrekturgrenzen dislozierten Frakturen klinisch-radiologische Kontrolle (ggf. mit Gipskeilung) nach 1 Woche. Ruhigstellung für 4 Wochen (präpubertäre Kinder) – 5 Wochen (pubertäre Kinder).

**Ergebnisse:**

Die konservative Behandlung von Frakturen der oberen Extremität gehört auch heute noch zum Goldstandard. Insbesondere bei pädiatrischen, aber auch bei erwachsenen Patienten kann durch eine korrekte Gipsruhigstellung unter überschaubarem Aufwand und mit gutem Kosten-Nutzen-Verhältnis eine korrekte Ausheilung der Fraktur bei guter Analgesie erreicht werden. Ein messbarer Parameter zur Kontrolle eines guten Gipses ist der Cast-Index.

## Abstract (en)

**Objective:**

Conservative treatment of stable fractures of the upper extremity in children.

**Indications:**

Undisplaced and age-tolerable displaced fractures of the hand, forearm, and elbow.

**Contraindications:**

Open fractures.

**Treatment options:**

Forearm splint/forearm cast for stable injuries to the radius or ulna. Long arm splint/long arm cast for injuries to the radius and ulna and after reduction of the forearm, as well as for stable, undisplaced injuries to the elbow. Intrinsic plus splint for injuries to the four fingers (excluding the thumb) and metacarpus.

**Further treatment:**

For stable injuries, immobilization for analgesia for 3–4 weeks. Clinical check after treatment. In the case of repositioned fractures or fractures displaced within the spontaneous correction limits, clinical–radiological control (if necessary, with cast wedging) after 1 week. Immobilization for 4 weeks (prepubertal children) or 5 weeks (pubertal children).

**Results:**

Conservative treatment of fractures of the upper extremity is still the gold standard today. In pediatric patients in particular, but also in adult patients, correct healing of the fracture with good analgesia can be achieved with manageable effort and a good cost–benefit ratio through correct cast immobilization. A measurable parameter for monitoring a good cast is the cast index.

## Vorbemerkungen

Verletzungen der oberen Extremitäten gehören zu den häufigen Verletzungen im Kindesalter. Auch heute noch ist, insbesondere in der Kindertraumatologie, die konservative Behandlung dieser Verletzungen für viele Frakturmuster der Goldstandard. Bei Kindern und Jugendlichen bestehen häufig stabile Verletzungen, welche je nach Lokalisation und Alter noch ein großes Korrekturpotenzial besitzen [1]. Die Technik des Gipsens ist keine neue Erfindung, wird jedoch oft nur wenig gelehrt. Daher möchten wir mit diesem Artikel die korrekte Technik der häufigsten Gipse der oberen Extremität nachvollziehbar darstellen.

In den meisten Kliniken wird heute mit einer Combicast-Technik gearbeitet. Hierbei wird der Gips aus einer Kombination von Softcast-Binden und Hardcast-Longuetten gefertigt. Diese Technik werden wir daher in diesem Kapitel auch vorstellen. Selbstverständlich ist aber auch eine Ruhigstellung der Verletzungen im Weißgips möglich.

## Behandlungsprinzip und -ziel

Wichtig bei der konservativen Versorgung von Verletzungen ist ein korrekter und gut sitzender Gips, welcher zum einen die Fraktur ruhigstellt, mit dem Hauptziel der Analgesie, zum anderen aber auch potenziell instabile Verletzungen zu halten vermag, um eine sekundäre Dislokation zu vermeiden. In Ausnahmefällen kann durch einen korrekten Gips und Gipskeilung auch eine Reposition von Angulationsfehlstellungen erreicht werden.

Die Verwendung von Schienen wird nur bei per se stabilen Verletzungen empfohlen.

Die Kenntnis der korrekten Gipstechnik ist eine Grundvoraussetzung für eine erfolgreiche konservative Therapie.

Weiterführende Gipstechniken, insbesondere die Gipskeilung, sind selbstverständlich auch an der oberen Extremität möglich. Die genaue Technik wird in einem weiteren Artikel dieser Ausgabe ausführlich behandelt, daher werden wir in diesem Kapitel nicht weiter darauf eingehen.

## Vorteile

- Keine Operation und somit kein Infektionsrisiko
- Kein Fremdmaterial mit der Notwendigkeit der Metallentfernung
- Narkose/Sedation nur bei gleichzeitiger Reposition notwendig
- Überschaubarer Materialaufwand

## Nachteile

- Risiko der sekundären Dislokation mit Notwendigkeit der operativen Versorgung
- Repetitive Nachkontrollen, ggf. Umgipsen oder Keilen im weiteren Verlauf notwendig
- Bei nicht per se stabilen Frakturen Röntgenverlaufskontrollen notwendig
- Risiko der Druckstellen bei nicht sachgerechter Technik und Polsterung
- Risiko des Kompartmentsyndroms bei zu engem und nicht gespaltenem Gips, bei Schwellung der Fraktur

## Indikationen

### Unterarmgips

- Wulstfrakturen des Unterarmes
- Undislozierte Frakturen des distalen Radius (metaphysär und epiphysär)
- Im alterstolerablen Bereich dislozierte, stabile Frakturen des distalen Radius (metaphysär und epiphysär). Die tolerierte Achsabweichung ist abhängig von der Lokalisation der Fraktur und dem Alter des Patienten. Diese Werte sind der entsprechenden Fachliteratur zu entnehmen

### Oberarmgips

- Undislozierte metaphysäre Frakturen des Unterarms
- Im alterstolerablen Bereich dislozierte Frakturen des distalen Unterarms
- Reponierte Frakturen des distalen Unterarms
- Undislozierte Unterarmschaftfrakturen
- Undislozierte/alterstolerabel dislozierte Frakturen des Ellbogens (Condylus radialis, Epicondylus ulnaris, undislozierte suprakondyläre Frakturen)
- Oberarmschaftfrakturen
- Postoperative Ruhigstellung und Analgesie

## Kontraindikationen

- Instabile Frakturen (relativ)
- Offene Frakturen
- Nicht reponierbare Monteggia-Frakturen
- Dislozierte Gelenkfrakturen
- Heute relative Kontraindikation → Grünholzfrakturen

## Patientenaufklärung

- Erklären der Vorteile der Gipsbehandlung sowie ggf. Abwägen der konservativen Behandlung gegen operative Alternativen
- Erklärung des Behandlungsablaufs und Behandlungsdauer sowie eventuell anstehender therapeutischer Interventionen (z. B. Reposition, Keilung)
- Hinweis auf das Risiko sekundärer Dislokationen mit dem eventuellen Risiko einer sekundären Operation mit Osteosynthese
- Hinweis auf Korrekturmechanismen (und Grenzen) des wachsenden Skeletts, insbesondere bei im alterstolerablen Bereich dislozierten Frakturen
- Hinweis auf Komplikationen der Gipsbehandlung (Druckstellen, Kompartmentsyndrom) und deren Symptome
- Informationen zum Umgang mit Problemen im Rahmen der Gipsbehandlung (leichte Schmerzen, Juckreiz, nasser Gips). Wir empfehlen hier die Erstellung eines Patienteninformationsblattes. Dieses sollte optimalerweise auch eine Telefonnummer zur Kontaktaufnahme bei akuten Problemen enthalten.
- Es ist darauf zu achten das nebst dem Kind *alle* Erziehungsberechtigen über die Behandlung und die Risiken aufgeklärt werden.

## Behandlungsvorbereitung

- Ausreichendes Personal sichern (Arzt, 1 Pflegekraft, bei Repositionen 2 Ärzte oder Pflegekräfte)
- Verfügbarkeit des Gipsraumes prüfen
- Material vorbereiten
- Wenn notwendig (z. B. bei Repositionen): Durchleuchtung installieren

## Material (Abb. 1)

- Stockinette oder Ähnliches in korrekter Weite (Achtung: zu kleine Schläuche schneiden ein, zu große Schläuche werfen Falten und verursachen Druckstellen)
- Polstermaterial (Watte oder Klebefilz für exponierte Stellen (z. B. Radiusstyloid, Epikondylen des Ellbogens)), Frotteestrumpf, für Allergiker stehen Polstermaterialen mit hohem Baumwollanteil oder bei Empfindlichkeit gegenüber Naturfaser aus synthetischen Fasern zur Verfügung
- Hardcast-Longuetten in 7,5 cm Breite (bei Kindern evtl. auch schmaler)
- Softcast-Binden in verschiedenen Breiten
- Mullbinden
- Kräftige Gipsschere, Gipsspreizer, oszillierende Gipssäge
- Handwarmes Wasser
- Durchleuchtung (bei instabilen Frakturen oder bei Repositionen)

**Abb. 1:**
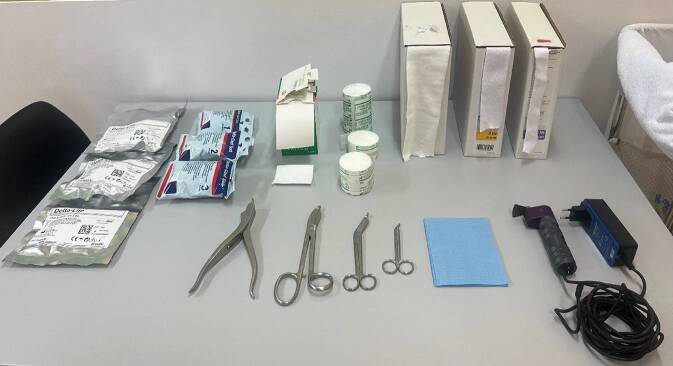
Standardmaterial zum Gipsen. Hardcast-Longuetten in verschiedenen Größen, Softcast-Bandagen in verschiedenen Breiten. Polstermaterial, Stockinette in verschiedenen Größen. Gipsspreizer, Gipsscheren in verschiedenen Größen, oszillierende Säge

## Anästhesie und Lagerung

**Abb. 2:**
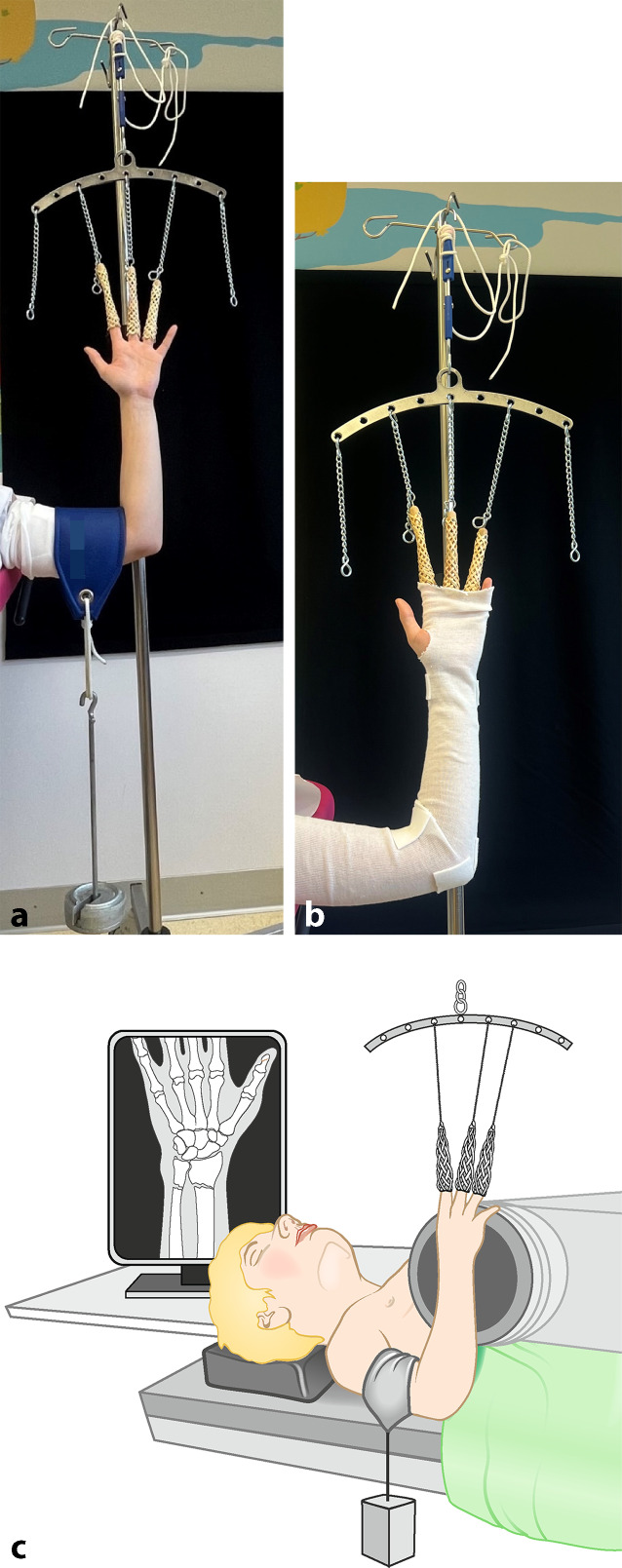
Gebrauch der Fingerextensionshülsen/Fingerstraps. Diese Installation bietet einem mehrere Vorteile. Zum einen kann der Arm durch Verwendung von Fingerstraps an mindestens 3 Fingern stabil ausgehangen werden. Der Stumpf ist vor dem Aufhängen anzulegen. Der Arm sollte so platziert werden, dass er von allen Seiten gut zugänglich und die optimale Gipsposition eingestellt ist. Der Gips kann so perfekt anmodelliert werden. Auch leicht abgekippte und beginnend instabile Frakturen können durch Applikation eines Gewichtes (je nach Größe des Kindes 1–3 kg, schrittweise steigern) in Achse gehalten oder reponiert werden (**a**). Weitere Vorteile sind, dass durch das Aushängen (auch ohne Gewicht) eine Assistenzperson zum Gipsen eingespart werden kann (**b**) und allein durch das Aushängen eine gewisse Analgesie erreicht wird, welche das Gipsen für die Patienten angenehmer macht. Diese Technik kann sowohl für Oberarmgipse als auch für Vorderarmgipse genutzt werden. Zusätzlich bietet sich die Möglichkeit der Installation eines C‑Arms zur Repositionskontrolle (**c**)

- Lagerung in Rückenlage, Arm zugänglich
- Ggf. Fingerextensionshülsen/Fingerstraps (Abb. 2)
- Ggf. Installation einer Durchleuchtung
- Eine Narkose ist nur bei Repositionen notwendig
- Analgosedierung je nach hausinterner Vorgabe

## Technik

### Grundlagen, Polsterung des Gipses

(Abb. 3)

**Abb. 3:**
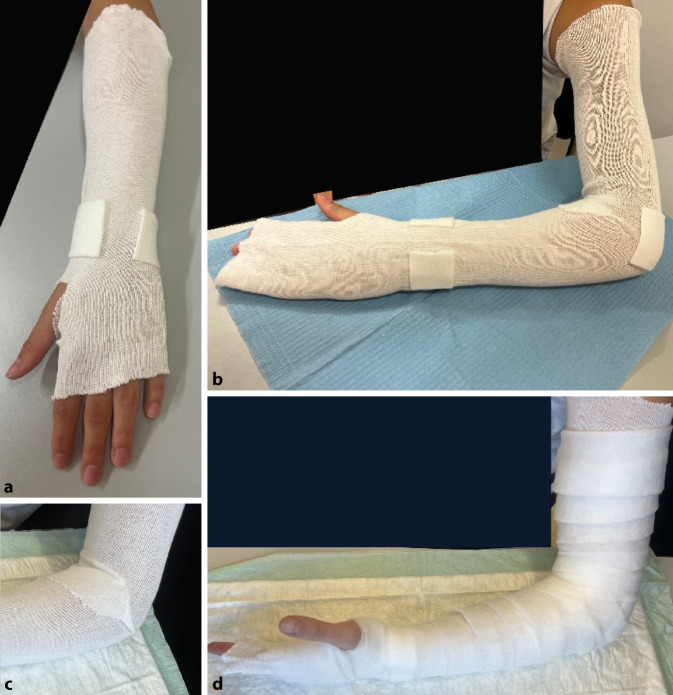
Der Gips wird den Patienten in aller Regel 4 bis 6 Wochen begleiten. Somit ist bei der Erstellung und Ausarbeitung des Gipses „Liebe zum Detail“ angebracht – dies sowohl bei der Vorbereitung und Polsterung als auch bei der Fertigstellung. Hierbei ist es unabhängig davon, ob es sich um eine Schiene oder einen Spaltgips oder einen zirkulären Gips handelt. Die Grundlagen der Polsterung und Ausarbeitung gelten für alle Modelle. *Ziel:* Das Ziel der Polsterung ist es, einen bequemen Gips anzulegen, welcher Druckstellen (insbesondere an Knochenvorsprüngen) verhindert, aber gleichzeitig eng genug anliegt, um eine Fraktur ausreichend zu stabilisieren. *Materialien:* In aller Regel wird zur Polsterung eines Gipses zuerst ein *dünner* Baumwollstrumpf angelegt, anschließend erfolgt die Polsterung von Knochenvorsprüngen mit einem Klebefilz insbesondere im Bereich des Processus styloideus ulnae und radii sowie der Epikondylen des Ellbogens und ggf. des Olekranons und der Ellbeuge und anschließend die gleichmäßige *dünne* Polsterung des gesamten zu gipsenden Bereichs mit Watte. Alternativ zur Watte stehen auch Frotteestrümpfe zur Verfügung, hier reicht in aller Regel das zusätzliche Abpolstern der Knochenvorsprünge. *Besonderheiten bei Allergikern:* Für Allergiker stehen spezielle Polstermaterialien zur Verfügung. Frotteestrümpfe enthalten einen hohen Anteil an Naturfasern. Dies kann von Vorteil sein. Bei Allergien gegen Naturfasern stehen auch rein synthetische Polstermaterialien zur Verfügung. Wir empfehlen, verschiedene Optionen vorrätig zu haben. Vorbereitung zur Gipsanlage bei einem Unterarmgips/Schiene (**a**) und Oberarmgips/Schiene (**b**). Der Strumpf sollte faltenfrei liegen. In der Ellbeuge sollte der Strumpf eingeschnitten werden, damit dort keine Falten entstehen (**c**). Prominente Knochenvorsprünge werden zusätzlich mit Klebefilz gepolstert, um Druckstellen zu vermeiden. Über den vorbereiteten Strumpf wird mit Watte gepolstert. Die Watte sollte dünn und gleichmäßig appliziert werden. Am *oberen* und *unteren* Abschluss kann etwas mehr Watte verwendet werden, um bei der Ausarbeitung später einen schön gepolsterten Rand zu erzeugen (**d**)

### Grundlagen zur Ausarbeitung des Gipses

(Abb. 4 und 5)

**Abb. 4:**
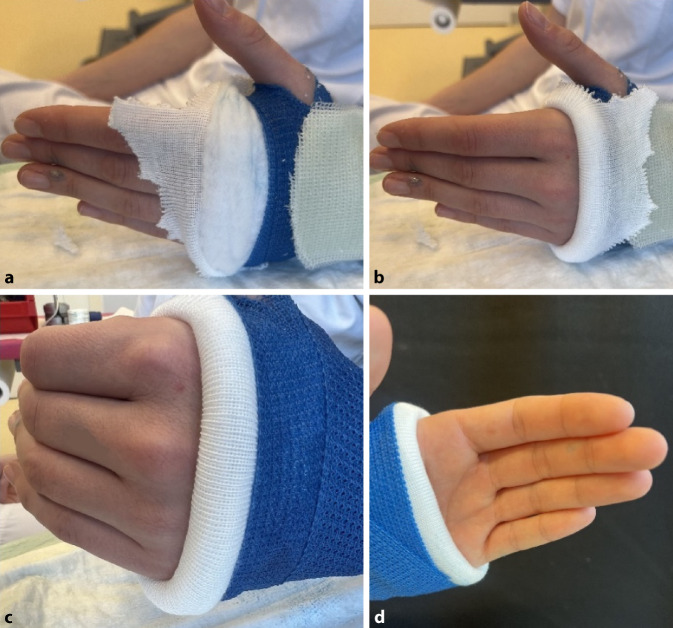
*Ziel:* Damit ein Gips auch langfristig bequem ist und es zu keinen Druckstellen kommt, sollten bei der Ausarbeitung ein paar Dinge beachtet werden. Um bei der Ausarbeitung einen weichen Rand zu erhalten, sollten der Strumpf und das Polster den eigentlichen Gips überragen (**a**). So kann nach Applikation der ersten Schicht Softcast sowie ggf. Anbringen der Verstärkung der Rand umgeschlagen werden, und es entsteht ein weicher Abschluss ohne die Gefahr von scharfen Kanten (**b**). Damit der Gips bequem ist und die Hand im Alltag brauchbar bleibt, sollte unbedingt darauf geachtet werden, dass die MCP-Gelenke frei bleiben (**c**). Im Bereich der Handfläche sollte der Gips daher maximal bis zur Hohlhandbeugefalte reichen (**d**)

**Abb. 5:**
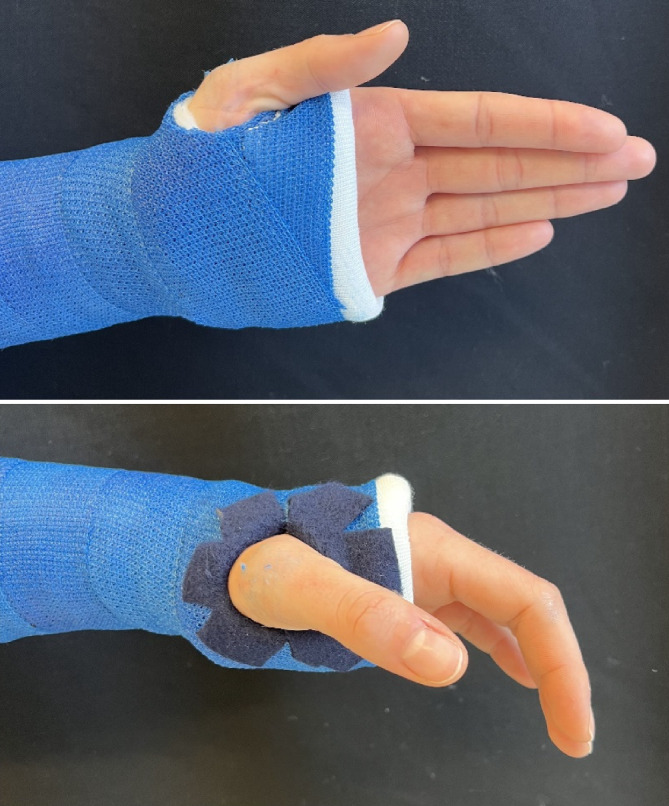
Bei der Ausarbeitung ist besonders der Daumen zu beachten. Das Daumengrundgelenk sollte frei sein (Abb. 5 oben). Die Ränder des ausgeschnittenen Daumens sind z. B. mit Klebefilz gut zu polstern (Abb. 5 unten), da durch das Ausschneiden scharfe Ränder entstehen können

### Ausarbeitung des Gipses – Cast-Index

(Abb. 6 und 7)

**Abb. 6:**
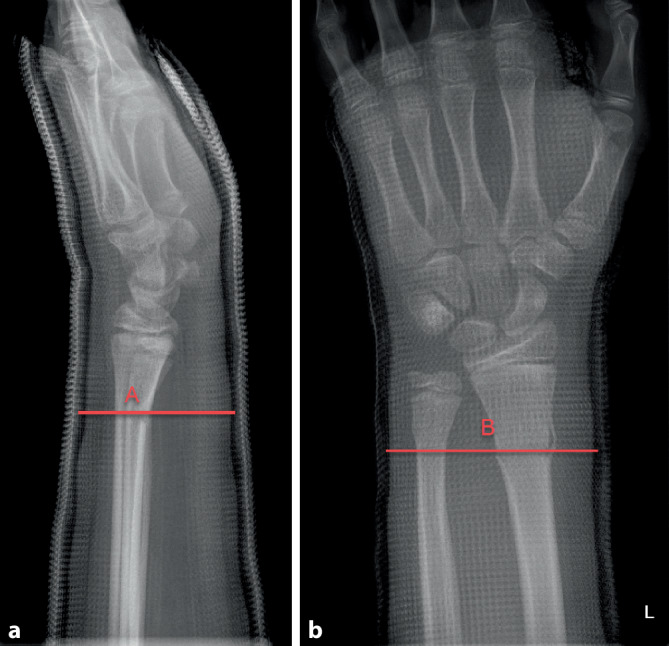
Damit ein Gips eine Fraktur zu halten vermag, muss er korrekt anmodelliert werden. Hierzu sollte das Wattepolster gleichmäßig verteilt sein und der Gips eng anliegen, ohne jedoch Druckstellen zu erzeugen. Das Anmodellieren des Gipses mit der flachen Hand, insbesondere im Frakturbereich, ist essenziell, erfordert jedoch etwas Übung und Erfahrung. Der Cast-Index ist ein Messwert, welcher zur Beurteilung des korrekten Anmodellierens herangezogen werden kann. *Bestimmung des Cast-Indexes.* Messung des Innendurchmessers (*A*) des Gipses auf Frakturhöhe im Seitenbild (**a**) geteilt durch den Innendurchmesser (*B*) des Gipses auf Frakturhöhe im a.-p. Bild (**b**). Optimal ist der Index bei 0,8 oder kleiner. Beachten Sie auch die durchgehende, gleichmäßige, aber nicht zu dick auftragende Polsterung

**Abb. 7:**
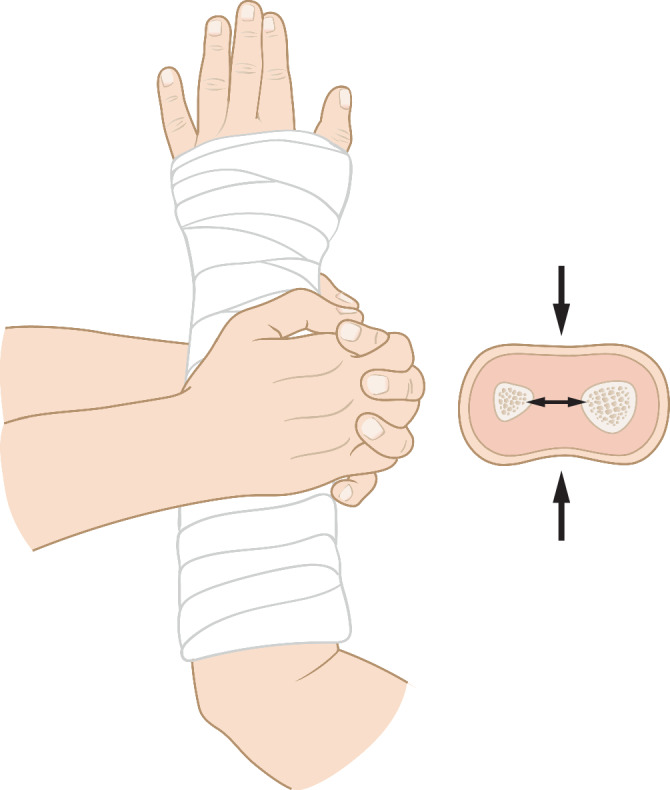
Durch Anmodellieren des Gipses mit flacher Hand wird der Gips in eine ovaläre Form gebracht. Hierdurch kommt es zum Aufspannen der Membrana interossea und somit zur zusätzlichen Stabilisierung der Fraktur. Es wird ein optimaler Cast-Index (s. Abb. 6a, b) erreicht. Des Weiteren liegt der Gips so dem Arm eng an und kann die Fraktur besser abstützen

### Gipsspaltung/Gipsentfernung

(Abb. 8 und 9)

**Abb. 8:**
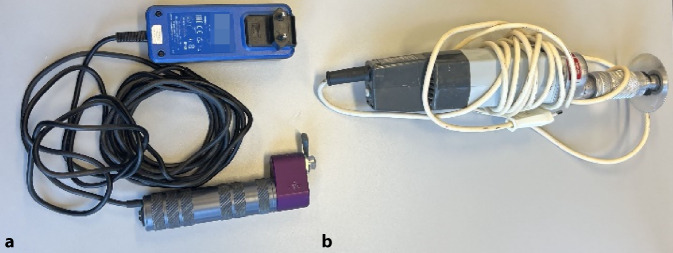
Ein Gips bei einer frischen Fraktur muss aufgrund des Risikos der Schwellung zu Beginn gespalten werden. Unter einer Gipsspaltung versteht man das Eröffnen des Gipses über die gesamte Länge und Aufschneiden des Polstermaterials bis auf die letzte Faser. Auch im späteren Verlauf der Behandlung kann eine Gipsspaltung bei zunehmenden Schmerzen im Gips notwendig werden. Bei zunehmenden Schmerzen im Gips ohne Besserung auf Schmerzmittel/Hochlagerung hat unverzüglich eine Gipsspaltung mit oben genannten Grundsätzen zu erfolgen. Wir empfehlen die Nutzung einer modernen Gipssäge mit oszillierendem/vibrierendem Sägeblatt (**a**) Bei älteren Modellen (**b**) ist die Verletzungsgefahr für die Haut größer. Des Weiteren sind sie sehr laut, die Verwendung eines Gehörschutzes für alle im Raum befindlichen Personen ist hier von Vorteil. Bei älteren Modellen kann außerdem die Staubbelastung für Patienten und Mitarbeitende relevant erhöht sein, sodass sich die Verwendung eines Mundschutzes und/oder Absaugung empfehlen. Des Weiteren werden zur Gipsentfernung Scheren in verschieden Größen benötigt (s. Abb. 1)

**Abb. 9:**
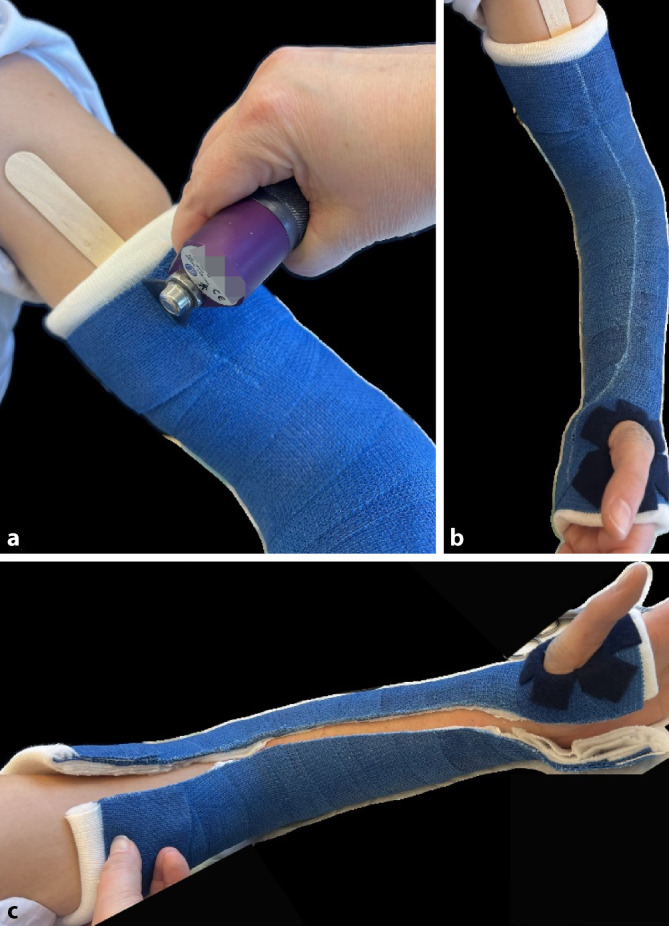
Wir empfehlen, vor der Verwendung den Kindern zu demonstrieren, dass die Gipssäge die Haut bei sachgerechtem Gebrauch nicht verletzen kann. Bei der Verwendung von Gipssägen entsteht Hitze. Daher sollte man beim Aufsägen des Gipses zwischendurch Pausen einlegen. Wenn man darauf achtet, den Gips im Bereich des Softcasts zu spalten, entsteht etwas weniger Hitze. Eine weitere Möglichkeit ist es, etwas unter die Säge zu legen, wir nutzen hierfür einen Holzspatel (**a**). Der Gips muss über die gesamte Länge aufgesägt werden (**b**). Bei instabilen oder reponierten Frakturen empfiehlt es sich, den Gips auf der der Dislokation abgewandten Seite zu spalten (z. B. palmare Spaltung bei initialer Dislokation des Vorderarms nach dorsal). Am Ende der Spaltung muss der Gips auf gesamter Länge geöffnet sein, und sämtliches Polstermaterial muss bis auf die letzte Faser aufgeschnitten sein (**c**). Zum Abschluss wird der Gips mit einer elastischen Binde wieder fixiert. Nach ca. 5 Tagen kann bei schmerzfreiem Patienten der Gipsschluss durch Umwickeln mit einer weiteren Binde Softcast erfolgen

#### Merke.

*Es gilt der Grundsatz: „Der Patient im Gips hat immer recht“* – im Zweifelsfall wird bei Schmerzen ein Gips gespalten (oder sogar gewechselt).

### Gipsschienen

#### Intrinsic-Plus-Schiene

(Abb. 10 und 11)

**Abb. 10:**
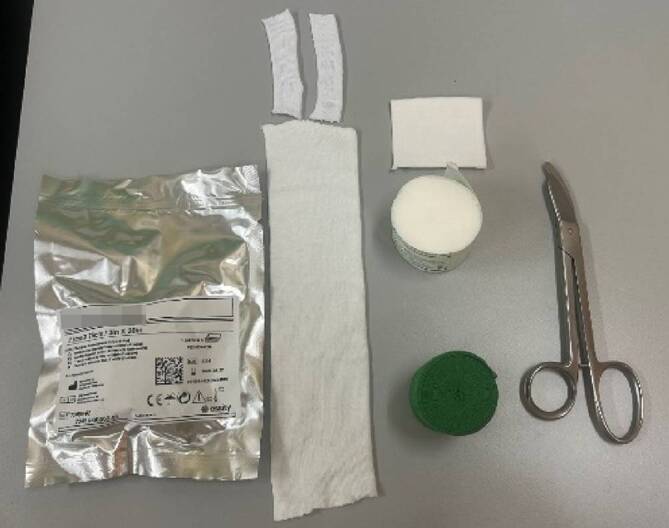
Benötigtes Material für die Intrinsic-Plus-Schiene sind Hardcast-Longuette, Polsterschlauch, Polstermaterial, Fingerschlauch oder Mullkompressen interdigital. *Indikationen: *Frakturen im Bereich der Grund- und Mittelphalanxknochen; Frakturen einer oder mehrerer Mittelhandknochen; Weichteilverletzung der Hand; postoperativ zur Ruhigstellung; präoperative Ruhigstellung von Verletzungen. Prinzipiell reicht eine Ruhigstellung des verletzten Fingers sowie der direkten Nachbarfinger. Bei kleineren Kindern empfehlen wir jedoch die vollständige Ruhigstellung der Dig. II–V, damit die Schiene nicht verrutscht

**Abb. 11:**
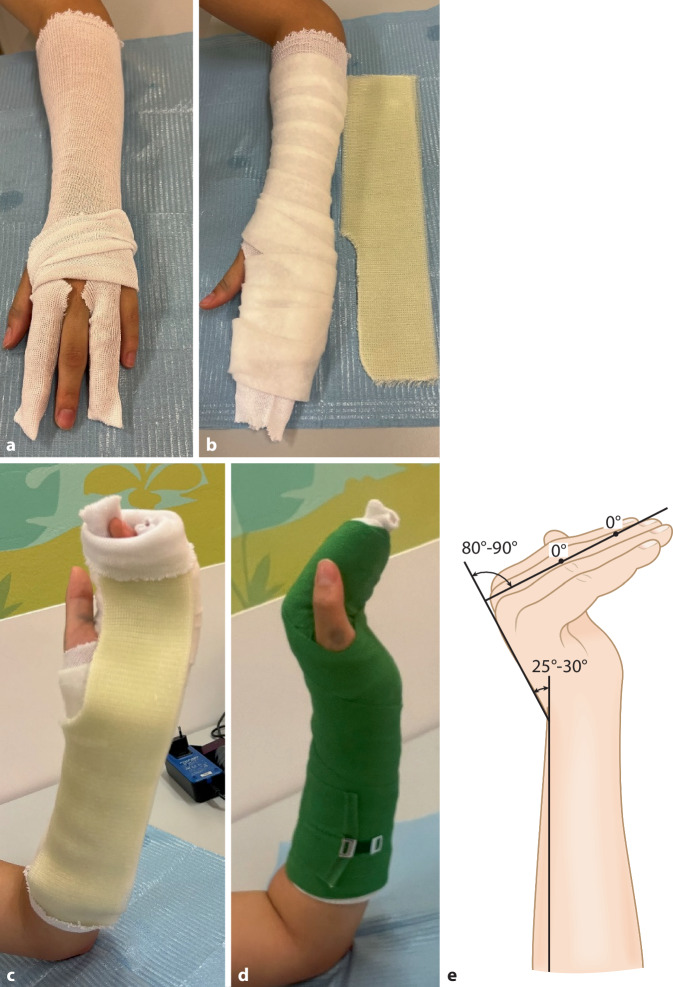
Vorbereiten der Hand durch Anlage des Polsterschlauches bis zur Ellbeuge. Um ein Schwitzen zwischen den Fingern zu vermeiden, empfehlen wir das (faltenfreie) Einlegen von Kompressen interdigital oder alternativ das Überziehen jedes zweiten Fingers mit einem kleinen Schlauchverband (**a**). Polstern der prominenten Knochenvorsprünge über Radius- und Ulnastyloid (s. auch Abb. 3a). Beachten sie auch die bereits vorbereitete Hardcast-Longuette. Für den Daumen wird die Schiene ausgeschnitten, um Druckstellen zu vermeiden (**b**). Die Longuette wird volar von 3 Fingerbreit distal der Ellbeuge nach proximal angelegt und gut anmodelliert (**c**). Zum Abschluss wird die Gipsschiene mit einer elastischen Binde fixiert (**d**). Die Hand wird in Intrinsic-Plus-Stellung zur Vermeidung von Kontrakturen: Handgelenk: 20–30° Dorsalextension, MCP 70–80° Flexion, PIP/DIP, gestreckt bis max. 10° Flexion (**e**)

### Unterarmschiene

(Abb. 12 und 13)

**Abb. 12:**
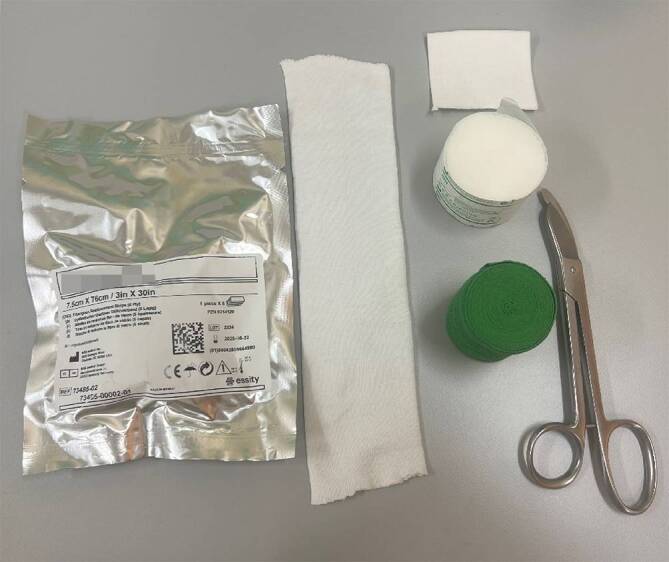
Benötigtes Material für Unterarmschienen sind Polsterschlauch, Hardcast-Longuette; Polstermaterial. *Indikationen: *stabile Verletzungen des Radius oder der Ulna; Prellungen; präoperative Ruhigstellung von Verletzungen des Unterarms

**Abb. 13:**
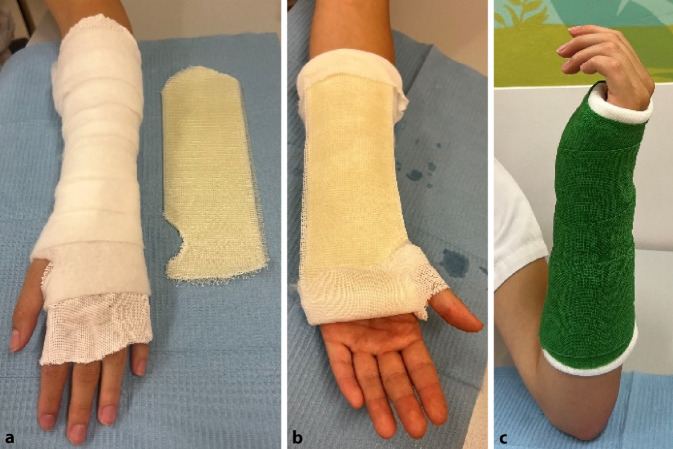
Anlegen des Polsterschlauchs bis kurz vor die Ellbeuge und Polstern mit Klebefilz (s. auch Abb. 3a, Polsterung) und/oder Watte (**a**), v. a. im Bereich der prominenten Knochenvorsprünge an Ulna- und Radiusstyloid. Beachte auch die bereits vorbereitete Hardcast-Longuette. Für den Daumen wird die Schiene ausgeschnitten. Anlegen der Longuette je nach Frakturtyp volar (nach volar eingestauchte Frakturen, undislozierte Frakturen) oder dorsal (nach dorsal eingestauchte Frakturen). Proximal reicht die Longuette bis max. 3 Querfinger distal der Ellbeuge (**b**). Handgelenkposition neutral bis leicht dorsalextendiert (10–15°). Fixieren mit einer elastischen Binde (**c**)

### Oberarmschiene

(Abb. 14 und 15)

**Abb. 14:**
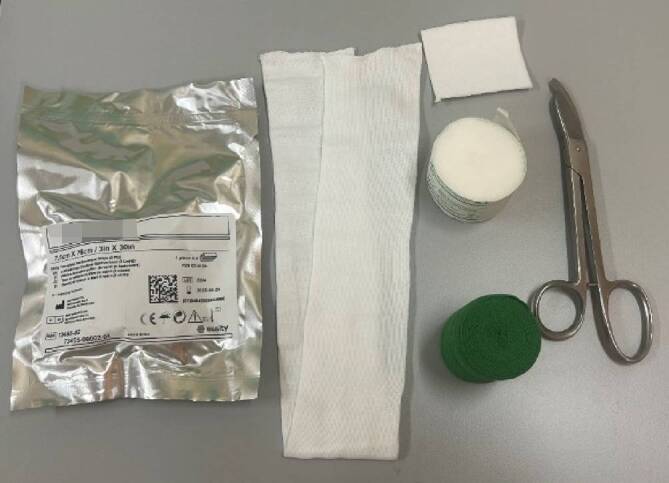
Benötigtes Material für die Oberarmschiene sind Polsterschlauch, Polstermaterial, Hardcast-Longuette. *Indikationen:* stabile Verletzungen des Radius und der Ulna; stabile Verletzungen des Radiuskopfes/-halses; stabile Verletzungen des Ellbogens; Prellungen; präoperative Ruhigstellung von Verletzungen des Unterarms oder Ellbogens

**Abb. 15:**
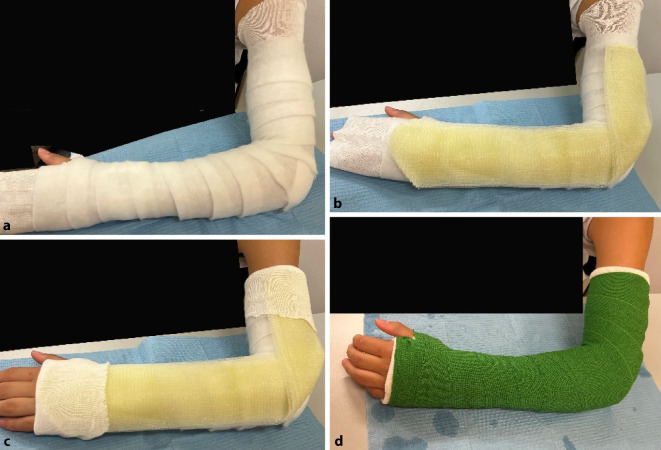
Im Gegensatz zur Vorderarmschiene werden bei der Oberarmschiene auch die Pro- und Supination ruhiggestellt. Anlegen des Polsterschlauches bis zur Schulter, Polstern mit Watte oder Klebefilz, v. a. im Bereich der prominenten Knochenvorsprünge an Ulna und Radiusstyloid, Epikondylen des Ellbogens (s. auch Abb. 3b). Polsterung mit Watte (**a**). Anlegen der Longuette L‑förmig an der Außenseite des Armes. Das Umklappen der Longuette zum L über dem Ellbogen erhöht die Stabilität. Tipp: Durch rundes Abschneiden der Enden der Longuette (**b**) werden scharfe Kanten vermieden. Das Polstermaterial sollte vor dem Umschlagen ca. 2 cm überragen, um einen weichen Rand der Schiene zu erhalten (**c**). Fixieren mit einer elastischen Binde, Handgelenkposition neutral oder leicht (10–15°) dorsalextendiert, Ellbogen 90° gebeugt (**d**)

### Zirkuläre Gipse

#### Hinweis.

Zur Vermeidung von Kompartmentsyndromen des Vorderarmes muss bei frischen Verletzungen mit hohem Risiko der Schwellung nach dem Aushärten ein Spalten des Gipses erfolgen (s. auch Abb. 9). Dies bedeutet, dass der Gips vollständig von proximal bis distal eröffnet wird, dies beinhaltet auch sämtliches zirkuläres Polstermaterial und den Strumpf.

#### Unterarmgips

(Abb. 16 und 17)

**Abb. 16:**
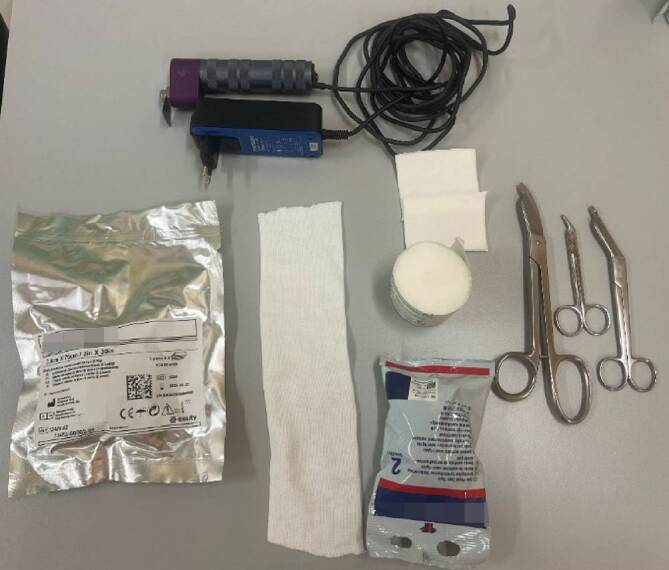
*Indikationen: *stabile Verletzungen des Radius oder der Ulna; undislozierte metaphysäre Frakturen des Radius oder der Ulna; im alterstolerablen Bereich dislozierte Frakturen des Radius oder der Ulna. *Benötigtes Material: *Polsterschlauch; Polstermaterial; Longuetten (Hardcast); Gipsbinde (Softcast); oszillierende Säge zum Spalten

**Abb. 17:**
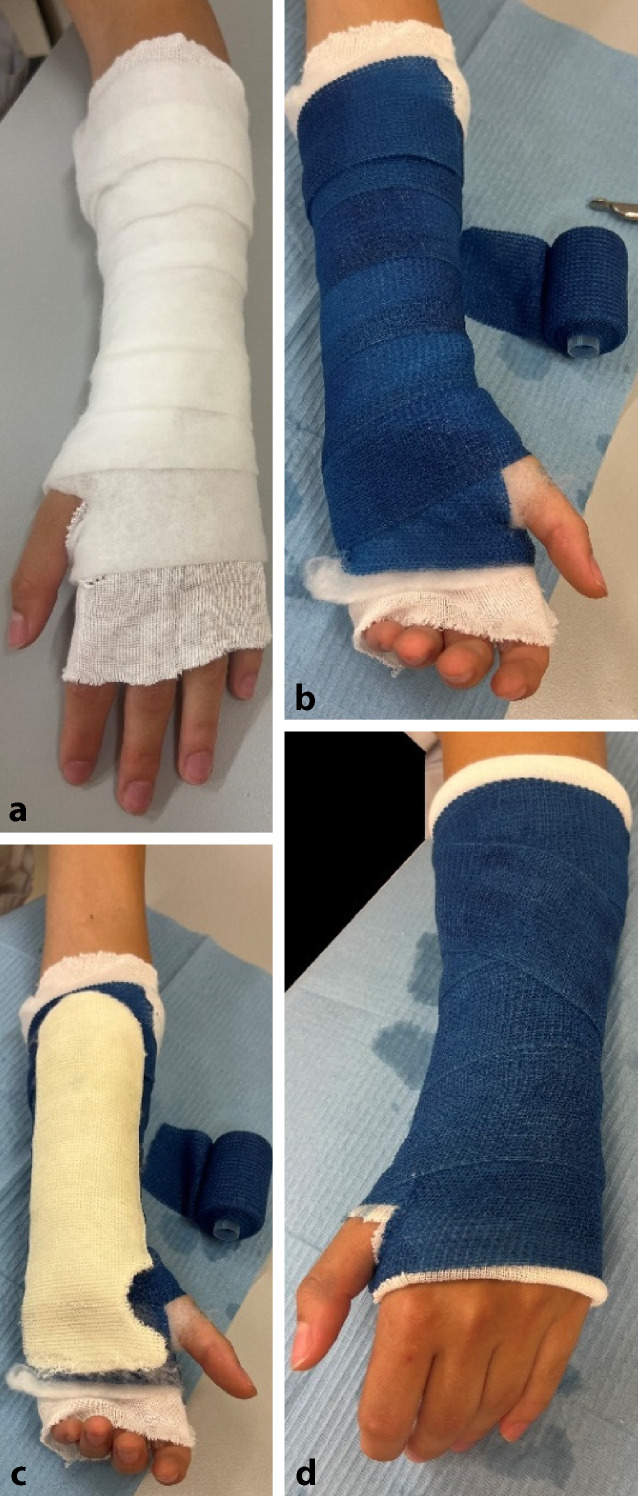
Anlegen des Polsterschlauchs bis kurz vor die Ellbeuge und Polstern mit Klebefilz (s. auch Abb. 3a, Polsterung) und/oder Watte (**a**), v. a. im Bereich der prominenten Knochenvorsprünge an Ulna- und Radiusstyloid. Das Polstermaterial wird zuerst mit einer Schicht Softcast umwickelt (**b**). Hier sollte darauf geachtet werden, das Polstermaterial proximal und distal ca. 2 cm überstehen zu lassen, damit der Gips am Ende einen weichen Rand erhält. Proximal reicht der Gips bis 3 Querfinger distal der Ellbeuge. Distal reicht der Gips bis in die distale Hohlhandbeugefalte, sodass die MCP frei beweglich bleiben (s. auch Abb. 4). Die Longuette wird, je nach Frakturtyp, volar (nach volar eingestauchte Frakturen, undislozierte Frakturen) oder dorsal (nach dorsal eingetauchte Frakturen) angelegt (**c**). Fixieren mit einer Softcast-Binde nach Umschlagen des Polsterschlauches (**d**). Auch hier sollte wieder darauf geachtet werden, dass das Daumengrundgelenk sowie die MCP und die Ellbeuge frei bleiben. Zum korrekten Anmodellieren des Gipses empfehlen wir des Weiteren bis zum Aushärten das Umwickeln mit einer nassen Mullbinde. So können die verschiedenen Schichten des Gipses sich gut miteinander verbinden und aushärten. Bei frischen Frakturen mit deutlichem Risiko der Schwellung Gipsspaltung unbedingt empfohlen. Bei der Spaltung des Gipses ist darauf zu achten, auch das Polstermaterial bis auf die letzte Faser zu spalten (s. auch Abb. 9) Selbstverständlich muss bei diesem Gips noch die Ausarbeitung des Daumens erfolgen (s. Abb. 5)

#### Oberarmgips

(Abb. 18 und 19)

**Abb. 18:**
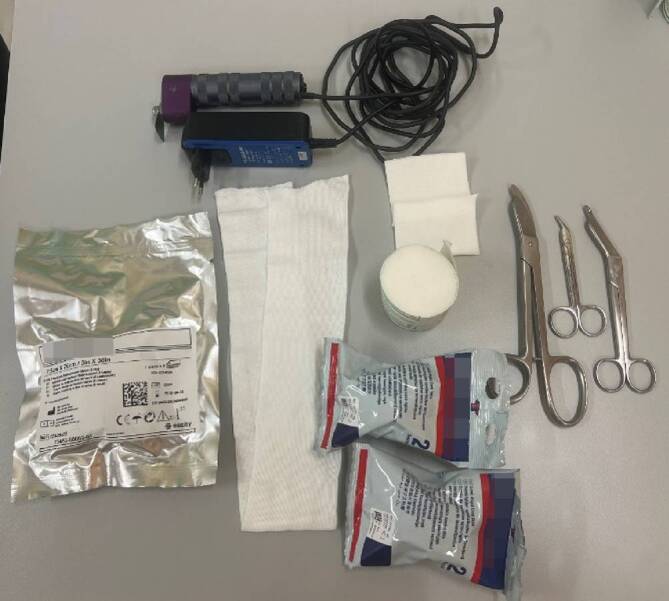
*Indikationen:* Verletzungen des Radius und der Ulna; nach Repositionen des Vorderarmes; Verletzungen des Ellbogens (nach Luxationen, epikondyläre und transkondyläre Frakturen); postoperative Ruhigstellung; Prellungen. Im Gegensatz zum Vorderarmgips wird beim der Oberarmgips auch die Pro- und Supination ruhiggestellt. *Benötigtes Material:* Polsterschlauch; Polstermaterial; Gips-Longuette (Hardcast); oszillierende Säge zum Spalten

**Abb. 19:**
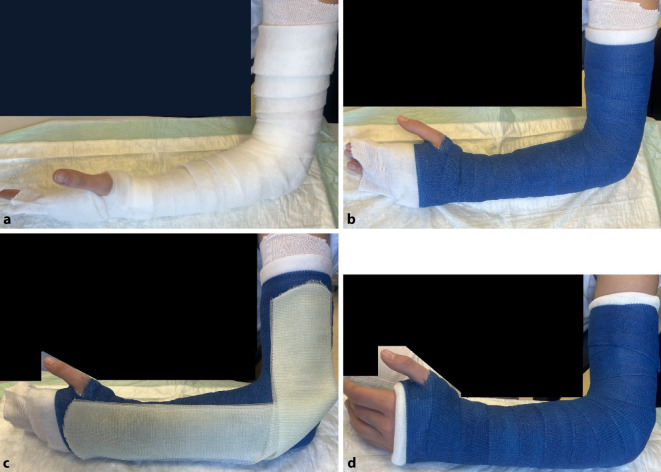
Anlegen des Polsterschlauches bis zur Schulter, Polstern mit Watte oder Klebefilz, v. a. im Bereich der prominenten Knochenvorsprünge an Ulna und Radiusstyloid, Epikondylen des Ellbogens (s. auch Abb. 3b). Polsterung mit Watte (**a**). Das Polstermaterial wird zuerst mit einer Schicht Softcast umwickelt (**b**). Hier sollte darauf geachtet werden, das Polstermaterial proximal und distal ca. 2 cm überstehen zu lassen, damit der Gips am Ende einen weichen Rand erhält. Proximal reicht der Gips bis 1 Handbreit distal der Axilla. Distal reicht der Gips bis in die distale Hohlhandbeugefalte, sodass die MCP frei beweglich bleiben (s. auch Abb. 4). Anlegen der Longuette L‑förmig an der Außenseite des Armes. Das Umklappen der Longuette zum L über dem Ellbogen erhöht die Stabilität (**c**). Der Polsterschlauch wird mit der überstehenden Watte umgeschlagen, es entsteht ein weicher Rand. Fixieren mit einer Softcast-Longuette. Handgelenkposition neutral. Ellbogen 90° gebeugt (**d**). Zum korrekten Anmodellieren des Gipses empfehlen wir des Weiteren bis zum Aushärten das Umwickeln mit einer nassen Mullbinde. So können die verschienen Schichten des Gipses sich gut miteinander verbinden und aushärten. Bei frischen Frakturen mit deutlichem Risiko der Schwellung Gipsspaltung unbedingt empfohlen. Bei der Spaltung des Gipses ist darauf zu achten, auch das Polstermaterial bis auf die letzte Faser zu spalten (s. Abb. 9) Selbstverständlich muss bei diesem Gips noch die Ausarbeitung des Daumens erfolgen (s. Abb. 5)

## Postoperative Behandlung

- Aufklärung über die möglichen Probleme der Gipsbehandlung (Druckstellen, Kompartmentsyndrom)
- Wenn vorhanden, Abgabe eines Merkblattes (ist zu empfehlen, ein solches in der Klinik bereitzustellen)
- Hochlagerung des Armes in den ersten Tagen empfohlen
- Gipsschluss nach 1 Woche
- Röntgenkontrolle nach 1 Woche nur bei potenziell instabilen Verletzungen und nach geschlossenen Repositionen
- Gipsbehandlung für 4 (präpubertäre Kinder) bis 5 (pubertäre Jugendliche) Wochen. Bei Kindern unter 3 Jahren reichen meist 3 Wochen Ruhigstellung aus
- Röntgenkontrolle nach Behandlungsabschluss bei initial instabilen Verletzungen und nach Repositionen

## Fehler, Gefahren, Komplikationen und ihre Behandlung

### Druckstellen.

Bei nicht sachgerechter Polsterung können unter dem Gips Druckstellen entstehen. An der oberen Extremität sind hier insbesondere die Epikondylen des Ellbogens und das Radius- und Ulnastyloid sowie die Ellbeuge zu nennen, jedoch können Druckstellen an jeglicher Lokalisation auftreten. Es ist daher bereits bei der Gipsanlage darauf zu achten das im Polstermaterial sowie im Gips keine Kanten oder Falten entstehen. Sollte es dennoch zu Druckstellen kommen, ist ein Gipswechsel mit besonderer Polsterung der Druckstellen (z. B. mit Hydrokolloidpflastern) notwendig.

### Schmerzen im Gips.

Insbesondere in den ersten Tagen nach Gipsanlage kann es zu Schmerzen im Gips kommen. Diese sind in der Regel entweder durch Druckstellen (s. oben) oder durch eine Schwellung im Gips bedingt. Des Weiteren kann auch die Fraktur selbst Beschwerden verursachen. Eine ausreichende Analgesie ist bei jeder Frakturbehandlung Grundvoraussetzung. Des Weiteren sollte, insbesondere in den ersten Tagen, die verletzte Extremität hochgelagert werden. Hierbei ist darauf zu achten das die Schwellung aus dem Arm ungehindert „abfließen“ kann, hierzu sollte die Hand also höher als der Ellbogen und Letzterer höher als die Schulter gelagert sein. Auch eine Kühlung durch den Gips ist hilfreich (Achtung vor Erfrierungen, kein direkter Kontakt von CoolPads und Haut). In den ersten Tagen nach einer Verletzung ist der Gips gepalten, sollten im weiteren Verlauf, nach Gipsschluss, Schmerzen auftreten, ist der Gips erneut zu spalten (inklusive aller einschnürenden Verbände, Schläuche und Polstermaterialien).

Es gilt der Grundsatz: *Der Patient im Gips hat immer recht!* Im Zweifelsfall muss bei persistierenden Beschwerden ein Gipswechsel erfolgen.

### Verdacht auf Kompartmentsyndrom.

Bei unstillbaren Schmerzen im Gips muss die sofortige Gipsspaltung inklusive aller einschnürenden Verbände, Schläuche und Polstermaterialien erfolgen. Bei ausbleibender Besserung sofortige Gipsentfernung sowie bei persistierenden Schmerzen operative Logenspaltung ohne Zeitverzug.

### Nasser Gips.

Sollte ein Gips nass geworden sein, kann versucht werden, den Gips mit der Kaltluftstufe des Föhns zu trockenen. Achtung: bei Verwendung von Heißluft besteht Verbrennungsgefahr. Bei ausbleibendem Erfolg sollte ein Gipswechsel erfolgen. Zur Vorbeugung eines nassen Gipses stehen verschiedene Möglichkeiten zur Verfügung: Altbekannt ist der Schutz des Gipses beim Duschen mit einem Plastiksack, hier haben wir die Empfehlung, diesen am oberen Rand noch mit Frischhaltefolie abzudichten, dies hat sich im Alltag bewährt. Inzwischen stehen selbstverständlich auch vorgefertigte Lösungen (Gipsspritzwasserschutz, Badeüberzug für Gipse) in Apotheken und Orthopädiefachgeschäften zur Verfügung. Auch wenn die Industrie hier eine Badetauglichkeit des Gipses verspricht, würden wir jedoch empfehlen, diese Überzüge nur als „Spritzwasserschutz“ zu sehen. Ein Hinweis: In der kalten Jahreszeit ist bei nass gewordenen Gipsen besondere Vorsicht geboten, es besteht die Gefahr der Erfrierungen. Hier sollte die Indikation zum Gipswechsel großzügig gestellt werden.

### Juckreiz unter Gips.

Durch Schwitzen, aber auch durch das Polstermaterial kann es zu Juckreiz unter dem Gips kommen. Die Gabe von Antihistaminika (z. B. Dimetindenmaleat-Tropfen) lindert den Juckreiz. Auf mechanische Maßnahmen (z. B. Stricknadeln/Lineal) sollte aufgrund der Verletzungsgefahr der Haut unbedingt verzichtet werden.

### Druckstellen an den Gipsrändern.

Scharfe Kanten, insbesondere von Hardcast-Materialien, können zu Druckstellen und Hautabschürfungen führen. Daher sind solche Kanten sorgfältig zu kontrollieren und ggf. zu kürzen und/oder mit Polstermaterial (z. B. Adheban™ Kantenschutzband) zu umkleben.

## Ergebnisse

Die konservative Behandlung von Frakturen der oberen Extremität gehört auch heute noch zum Goldstandard. Insbesondere bei pädiatrischen, aber auch bei erwachsenen Patienten kann durch eine korrekte Gipsruhigstellung unter überschaubarem Aufwand und mit gutem Kosten-Nutzen-Verhältnis eine korrekte Ausheilung der Fraktur bei guter Analgesie erreicht werden.

Verschiedene Faktoren beeinflussen das Behandlungsergebnis nach Gipsbehandlung. Diese Einflussfaktoren sind insbesondere für die klassischen distalen Vorderarmfrakturen mehrfach untersucht. Zu den prädiktiven Faktoren zählen zum einen frakturspezifische Faktoren, so zeigen sich schlechtere Ergebnisse bei Fraktur beider Vorderarmknochen oder bei vollständiger Dislokation der Fraktur, zum anderen technische Faktoren wie das anatomische Repositionsergebnis [2]. Des Weiteren beeinflussen auch gipsspezifische Faktoren den Erfolg der Behandlung. Zu diesen Faktoren zählt der Cast-Index [3].

Der Cast-Index ist definiert als der innere Durchmesser des Gipses auf Frakturhöhe im Seitenbild geteilt durch den inneren Durchmesser des Gipses auf Frakturhöhe im a.-p. Bild (s. Abb. 6 und 7). Er ist ein Messwert zur Kontrolle des korrekten Anmodellieren des Gipses [4] Ein Cast-Index unter 0,80 gilt als guter prognostischer Faktor mit geringerem Risiko der sekundären Dislokation bei Vorderarmfrakturen [3, 4]. Die Datenlage zum Cast-Index ist zwar bis heute sehr dünn und heterogen, mehrere Studien konnten jedoch den positiven Effekt für den Cast-Index nachweisen [2]. Damit kann dieser Parameter zur Beurteilung eines „guten Gipses“ im Alltag unserer Meinung nach durchaus hilfreich sein.

## Data Availability

Die in dieser Studie erhobenen Datensätze können auf begründete Anfrage beim Korrespondenzautor angefordert werden.
